# Mechanisms of Cigarette Smoke Effects on Human Airway Smooth Muscle

**DOI:** 10.1371/journal.pone.0128778

**Published:** 2015-06-15

**Authors:** Mark E. Wylam, Venkatachalem Sathish, Sarah Kay VanOosten, Michelle Freeman, David Burkholder, Michael A. Thompson, Christina M. Pabelick, Y. S. Prakash

**Affiliations:** 1 Department of Internal Medicine, Mayo Clinic College of Medicine, Rochester, Minnesota, United States of America; 2 Department of Pediatrics, Mayo Clinic College of Medicine, Rochester, Minnesota, United States of America; 3 Department of Anesthesiology, Mayo Clinic College of Medicine, Rochester, Minnesota, United States of America; 4 Department of Physiology & Biomedical Engineering, Mayo Clinic College of Medicine, Rochester, Minnesota, United States of America; University of Debrecen, HUNGARY

## Abstract

Cigarette smoke contributes to or exacerbates airway diseases such as asthma and COPD, where airway hyperresponsiveness and airway smooth muscle (ASM) proliferation are key features. While factors such as inflammation contribute to asthma in part by enhancing agonist-induced intracellular Ca^2+^ ([Ca^2+^]i) responses of ASM, the mechanisms by which cigarette smoke affect ASM are still under investigation. In the present study, we tested the hypothesis that cigarette smoke enhances the expression and function of Ca^2+^ regulatory proteins leading to increased store operated Ca^2+^ entry (SOCE) and cell proliferation. Using isolated human ASM (hASM) cells, incubated in the presence and absence cigarette smoke extract (CSE) we determined ([Ca^2+^]i) responses and expression of relevant proteins as well as ASM proliferation, reactive oxidant species (ROS) and cytokine generation. CSE enhanced [Ca^2+^]i responses to agonist and SOCE: effects mediated by increased expression of TRPC3, CD38, STIM1, and/or Orai1, evident by attenuation of CSE effects when siRNAs against these proteins were used, particularly Orai1. CSE also increased hASM ROS generation and cytokine secretion. In addition, we found in the airways of patients with long-term smoking history, TRPC3 and CD38 expression were significantly increased compared to life-long never-smokers, supporting the role of these proteins in smoking effects. Finally, CSE enhanced hASM proliferation, an effect confirmed by upregulation of PCNA and Cyclin E. These results support a critical role for Ca^2+^ regulatory proteins and enhanced SOCE to alter airway structure and function in smoking-related airway disease.

## Introduction

Cigarette smoke contributes to or exacerbates airway diseases, such as asthma and COPD, where airway hyperresponsiveness (AHR) and airway smooth muscle (ASM) proliferation are key features. Importantly, even brief exposure to secondhand cigarette smoke affects lung function and inflammatory cytokine synthesis in humans [1]. However, the precise mechanisms by which cigarette smoke affects airway structure and function are still under investigation.

In ASM, changes in intracellular Ca^2+^ concentration ([Ca^2+^]_i_) regulate contraction and cell proliferation, and enhancement of both may contribute to the key features of asthma or COPD. Animal data indicates that sidestream cigarette smoke exposure increases ASM responsiveness to ACh and high K^+^ depolarization in rats: an effect that may be due to augmented RhoA-mediated Ca^2+^ sensitization [2]. We recently showed that thymic stromal lymphopoietin increases [Ca^2+^]_i_ responses of hASM to agonist [3]. In this regard, the mechanisms underlying AHR and ASM proliferation may indeed be triggered by increased [Ca^2+^]_I,_ mediated by agonist (e.g. ACh, histamine [4]) stimulated Ca^2+^ release from sarcoplasmic reticulum (SR) triggered by inositol 1,4,5-triphosphate (IP3) or activation of ryanodine receptor (RyR) channels possibly by cADPR via CD38 activation [5,6,7,8,9,10,11] or by plasma membrane Ca^2+^ influx. While a number of influx mechanisms exist in ASM, replenishment of SR calcium stores via store-operated Ca^2+^ entry, (SOCE) is thought to be particularly important in AHR. Previously, we have shown that *in vitro* exposure of human ASM (hASM) cells to TNF-α, a potent proinflammatory cytokine, augments agonist-elicited Ca^2+^ mobilization and SOCE: effects associated with significant increases in the expression of membrane-bound Ca^2+^ regulatory proteins TRPC3 and CD38. Whether similar Ca^2+^ regulatory mechanisms are at play in cigarette smoke effects on hASM is not known. In the present study, we examined the hypothesis that cigarette smoke extract (CSE) augments agonist-elicited [Ca^2+^]_i_ responses by enhanced Ca^2+^ regulatory protein expression (particularly SOCE), and furthermore increases hASM proliferation.

## Materials and Methods

DMEM, antibiotic/antimycotic mixture and fura-2 were obtained from Invitrogen, Carlsbad, CA. All fine chemicals were purchased from Sigma-Aldrich, St. Louis, MO. Cyclopiazonic acid was purchased from Calbiochem, La Jolla, CA.

### Human Bronchial Tissue and hASM Cells

The techniques for isolation of hASM cells have been previously described [12,13]. Briefly. pathologically normal lung areas were dissected from post-surgical samples of patients undergoing pneumonectomies or lobectomies for focal, non-infectious disease (approved by Mayo’s Institutional Review Board and considered not Human Subjects Research; accordingly, patient consent was waived). IRB-approved protocols permitted initial review of patient histories, allowing us to exclude smokers, asthmatics or patients with COPD. Therefore, we were able to avoid the confounding effect of variable chronic inflammation and/or smoke exposure on the parameters studied. Following tissue isolation, samples were completely de-identified for storage and subsequent usage. In this study, we used airways from both males (6) and females (5).

From the lung samples, 3^rd^ to 6^th^ generation bronchi were dissected, immersed in ice-cold Hanks balanced salt solution (in mM, Ca 2.25; Mg 0.8; glucose 12; pH 7.4; HBSS) aerated with 100% O_2_ and the epithelium was removed by abrasion. The ASM cells were isolated using enzymatic dissocation as previously described [12,13]. Cells were seeded into culture flasks and maintained under standard culture conditions for up to passage 5 of subculture. Maintenance of ASM phenotype was confirmed by immunohistochemistry and RT-PCR or Western analysis for smooth muscle alpha-actin, myosin and other smooth muscle markers. ASM cells were exposed to serum-free medium for at least 24 h before treatment with CSE or experimentation.

### CSE preparation

The technique for CSE preparation based on a modification of the method of Blue and Janoff [14] has been described previously [3,15]. Briefly, aqueous CSE was prepared via a smoking apparatus to which smoke from a Kentucky 1RF4 cigarette was slowly bubbled into DMEM/12 (1 cigarette/10 ml serum free medium). CSE was prepared fresh daily for experimentation. Nicotine and other metabolite concentrations in the CSE were analyzed using LC-MS [15]. The literature describes a broad range of CSE concentrations, but in pilot studies, we determined the effect of CSE on ASM cellular viability (trypan blue or propidium iodide exclusion) and found that ≥5% CSE concentration produced significant cell death, especially with prolonged exposures. Accordingly, we used lower concentrations (1 or 2%) to determine modulatory effects of CSE. A time period of 24 h cell incubation with CSE was selected based on previous observation of altered protein expression within that time frame.[15]

### Western analyses

Standard denaturing SDS-PAGE techniques were applied to hASM cell lysates. Proteins were transferred onto PVDF membrane, blocked with 5% non-fat milk and probed with antibodies of interest. Primary antibodies raised in mouse or rabbit against TRPC3, ß-Actin, CD38, STIM1, Orai1, GAPDH, PCNA, and Cyclin E were all obtained from Santa Cruz Biotechnology (Santa Cruz, CA, USA). Protein detection was performed using LiCor IR-conjugated secondary anti-mouse or anti-rabbit antibodies, detected with a LiCor Odyssey gel documentation system.

### siRNA Transfection

We have previously described siRNA knockdown of proteins including TRPC3, CD38, Orai1 and STIM1 in hASM [12,16]. Transfection was achieved using 20 nM siRNA and Lipofectamine 2000 (Invitrogen) in DMEM F-12 lacking FBS and antibiotics. Fresh growth medium was added after 6 h and cells analyzed after 48 h. For each of TRPC3, CD38, STIM1 and Orai1, 25-bp duplex siRNAs were used as previously described (Invitrogen, Carlsbad, CA for TRPC3 and CD38, Ambion, Austin, TX, USA for STIM1 and Orai1). As a negative control, the Silencer Negative Control #1 (Ambion) was used. Knockdown efficacy and specificity was verified by Western blot analysis.

### [Ca^2+^]_i_ imaging

The techniques for [Ca^2+^]_i_ measurements in hASM using fluorescent indicators have also been previously described [12,17]. Briefly, cells plated on 8-well Labtek glass-bottomed chambers were incubated in 5μM Fura-2AM (Invitrogen, Eugene, OR), washed and perfused with HBSS. Fluorescence was recorded using a real-time imaging system (Metafluor; Molecular Devices, Sunnyvale, CA; Roper Scientific 12 bit camera system) on an inverted Nikon Diaphot microscope (40X/1.3NA objective lens), with [Ca^2+^]_i_ calculated from the 340/380 ratios based on an *in vitro* calibration [18].

### Store-operated Ca^2+^ entry

The techniques for SOCE measurements in ASM cells have also been previously described [12,19]. ASM cells were initially perfused with HBSS and baseline [Ca^2+^]_i_ levels recorded. Extracellular Ca^2+^ was then removed using nominally Ca^2+^-free HBSS, followed by SR Ca^2+^ depletion using 10 μM cyclopiazonic acid (CPA; inhibitor of SR Ca^2+^ ATPase), and rapid reintroduction of extracellular Ca^2+^ in the continued presence of CPA to trigger SOCE.

### Measurement of ROS generation

hASM cells were pre-exposed to vehicle or 3 mM N-acetylcysteine and then to 1% CSE for 4h. Cells were then loaded with 5μM of dichlorodihydrofluorescein diacetate (H2DCFDA, Calbiochem, San Diego, CA, USA) for 30 min, washed, and immediately analyzed for fluorescence intensity using a FlexStation3 microplate reader (Molecular Devices).

### Measurement of TNF-α concentration

hASM cells were treated with 1% CSE for 24h in the presence or absence of NAC in 96-well plates. The supernatants were collected and analyzed for secreted TNF-α using ELISA kits (BioSource; Camarillo, CA, USA) according to the manufacturer's instructions.

### Determination of Cell Proliferation

ASM cells were grown to approximately 50% confluence in 96-well culture plates, and were exposed to serum- and antibiotic-free medium for 24 h. Proliferation of ASM cells over 48h was assayed using the CyQuant NF dye (Invitrogen) as described previously [20]. Dye calibrations were performed empirically using different cell counts and used to obtain estimates of baseline proliferation and with drug exposures over the 48 h period. Extent of proliferation was normalized to baseline (i.e. time zero).

### Statistical analysis

Statistically significant differences between cell groups were analyzed using one- or two-way ANOVA as appropriate (control vs. TNFα, non-transfected vs. transfected, etc.). Multiple comparison tests (Tukey or Scheffe) were employed as appropriate. Experiments involved hASM cells obtained from at least 6 different patients. A value of P< 0.05 was considered significant. All results are expressed as mean ± SEM.

## Results

### Cigarette smoking increases expression of CD38 and TRPC3 in hASM

**Fig 1:**
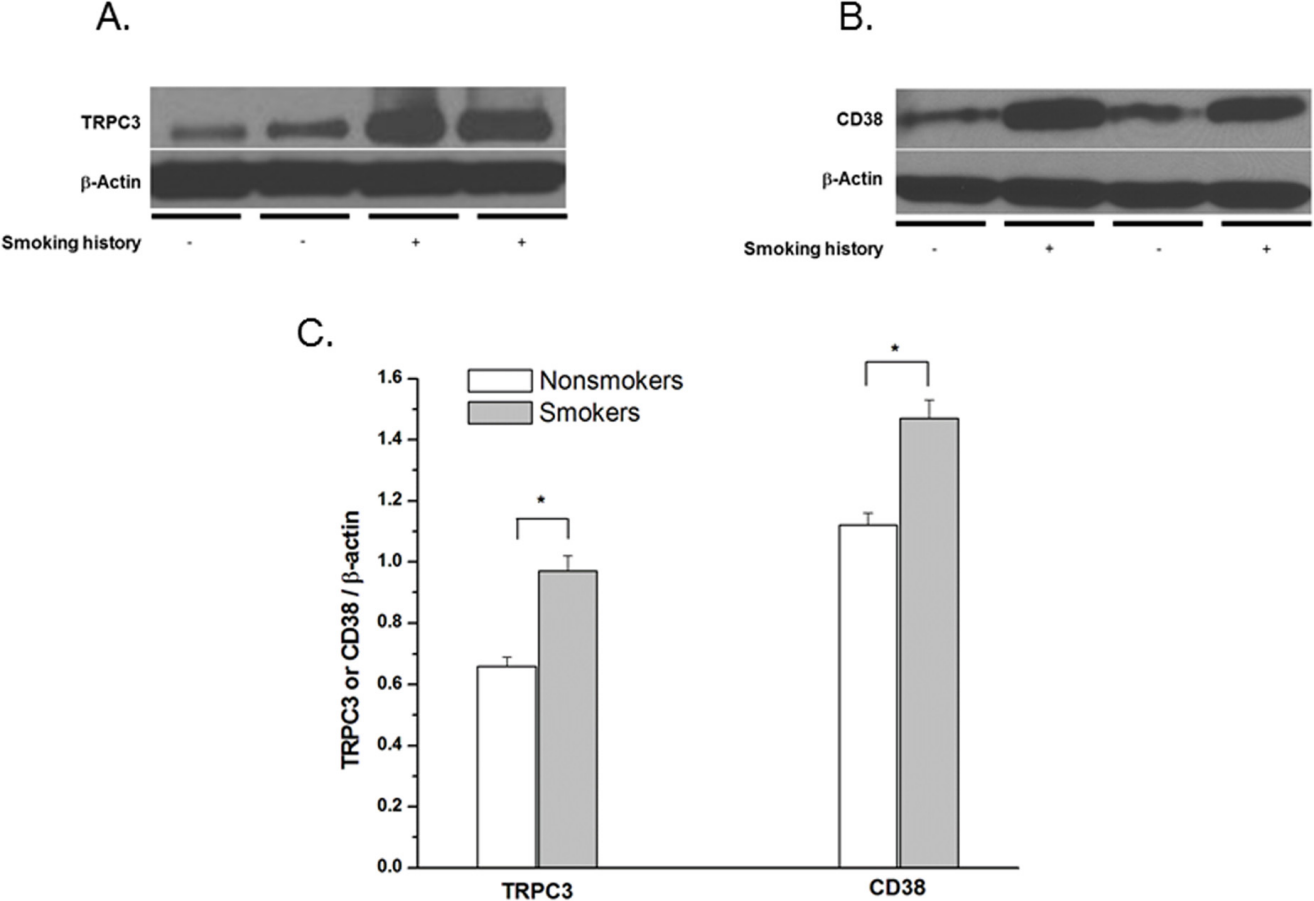
Cigarette smoking increases expression of CD38 and TRPC3 in hASM. Effect of strong smoking history (25 pack-years or greater) compared to lifelong non-smokers on expression of TRPC3 (A) and CD38 (B) from homogenized bronchial tissue obtained from surgical patients. (C) Densitometry values are mean ± SE and are normalized to housekeeping protein (ß-actin). n = 8 surgical specimens for each condition. * = P < 0.001.

Western blot analysis of lysates from epithelium-denuded whole hASM tissue of human bronchi showed significant increases in protein expression (normalized to ß-actin) of both TRPC3 (Fig 1A) and CD38 (Fig 1B) from patients with significant smoking history (> 25 pack years) when compared to life-time non-smokers (p < 0.02 for either comparison; n = 8, summarized in Fig 1C).

### CSE increases Ca^2+^ regulatory protein expression in hASM

**Fig 2:**
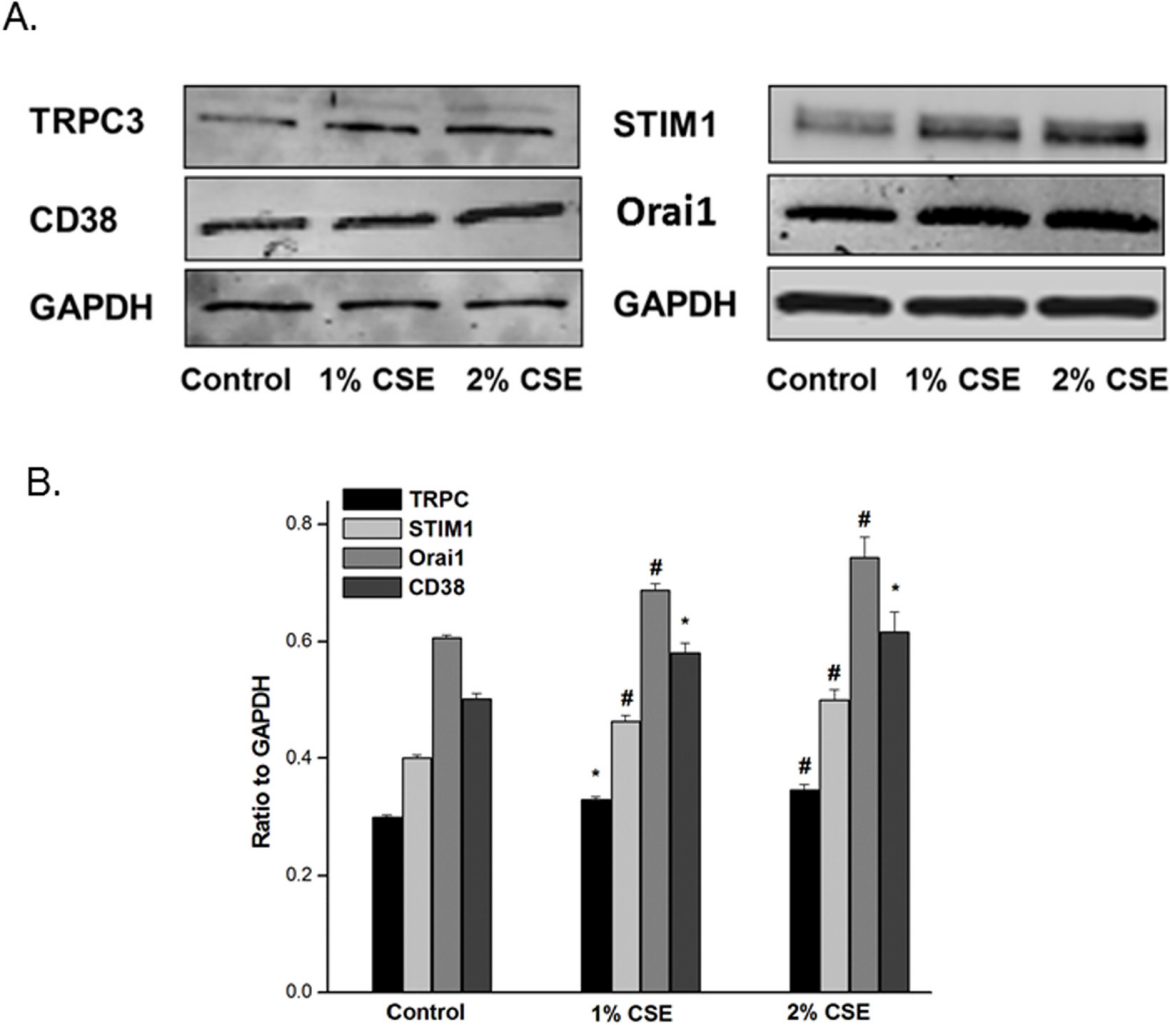
CSE increases Ca^2+^ regulatory protein expression in hASM. Effect of overnight CSE on CD38, TRPC3, STIM1 and Orai1 expression in hASM cell lysates. Western blot analysis shows significant increases in protein expression in both CSE groups when compared to respective control. (A) Cumulative results with ratio to GADPH. N = 8–16 in each group, * < 0.0005, # < 0.0001. (B) Representative Western blots showing CSE incubation increases calcium regulating proteins in hASM.

Western blot analysis (Fig 2A) of cell lysates from hASM cells exposed overnight to 1% or 2% CSE showed concentration-dependent increase in the [Ca^2+^]_i_ regulatory proteins TRPC3, CD38, STIM1, and Orai1, compared to vehicle treated control cells (P<0.05; n = 8; Fig 2B).

### CSE enhances [Ca^2+^]_i_ responses in hASM

**Fig 3:**
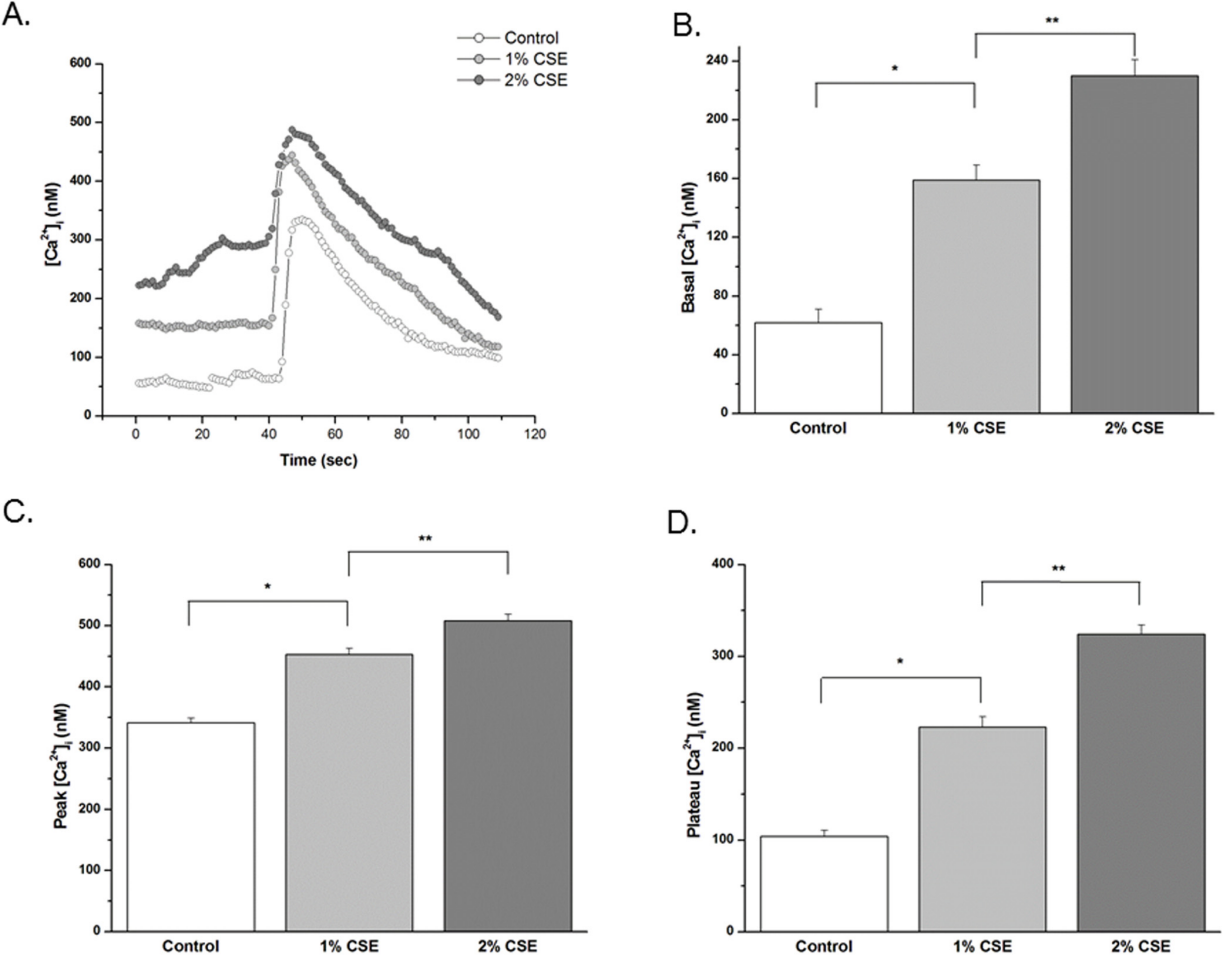
CSE enhances [Ca^2+^]_i_ responses in hASM. Effect of *in vitro* CSE incubation on agonist [Ca^**2+**^]_**i**_ responses. hASM cells exposed overnight to vehicle (control), 1, or 2% CSE, were loaded with fura-2AM, and visualized using real-time fluorescence microscopy. Typical tracings (A) elicited by maximal (10 μM) BK. (B) Cumulative data of CSE at 1% and 2% eliciting increased basal, and maximal (10 μM) BK-elicited peak and plateau [Ca^**2+**^]_**i**_ responses. (C) Exposure to submaximal (1 μM) histamine elicited a characteristic transient increase in [Ca^**2+**^]_**i**_ in all cell groups. Compared with vehicle control, ASM cells exposed to CSE produced higher peak [Ca^**2+**^]_**i**_ response.

In fura 2AM-loaded hASM cells not incubated with CSE, baseline [Ca^2+^]_i_ levels ranged from 35 to 84 nM (n = 228, derived from 7 patient samples, 25–40 cells/sample). Acute exposure to 10 μM bradykinin (maximal response) produced a typical transient [Ca^2+^]_i_ elevation followed by a lower plateau (Fig 3A). Overnight exposure to 1% and 2% CSE increased basal, peak and plateau [Ca^2+^]_i_ values (Fig 3B). A similar profile of CSE enhanced responses was noted for submaximal response to 1 μM histamine (peak [Ca^2+^]_i_ response data shown in Fig 3C).

### CSE enhances SOCE in hASM

**Fig 4:**
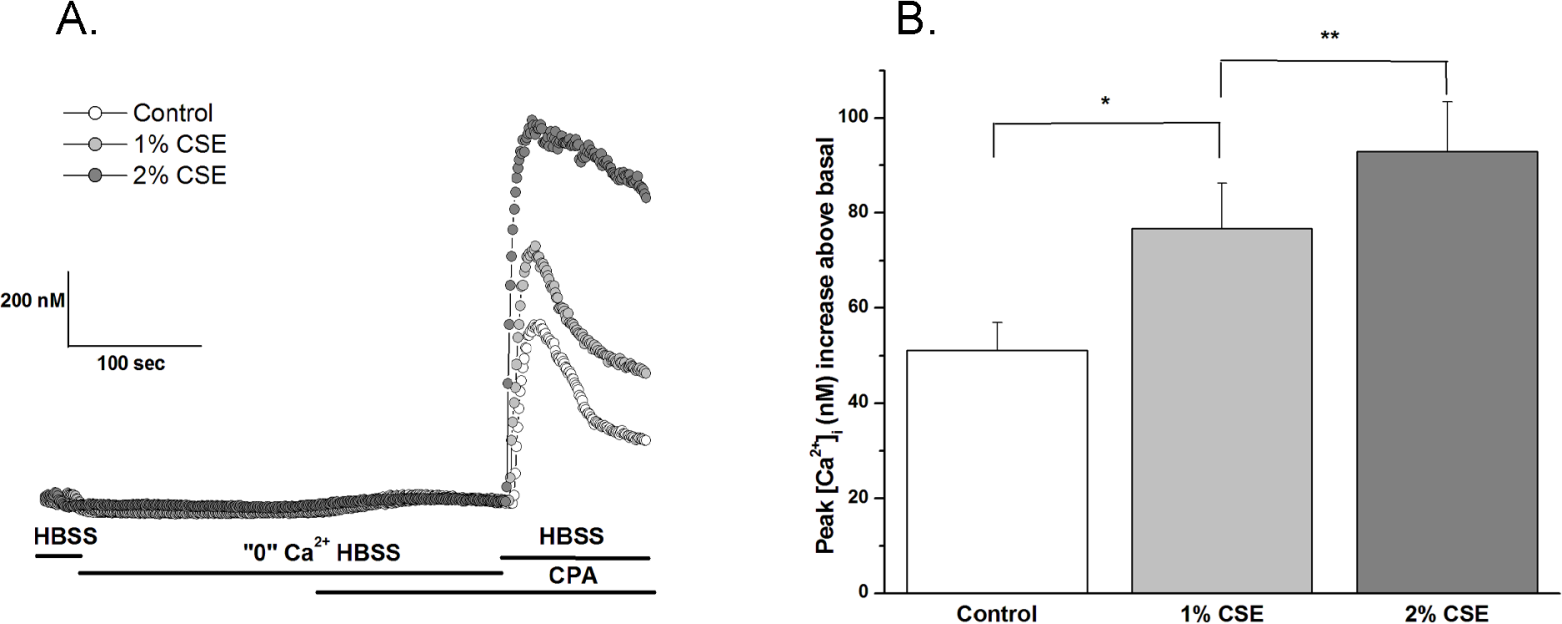
CSE enhances SOCE in hASM. Effect of CSE on store operated Ca^**2+**^ entry on hASM. In the continued presence of Nifedipine (1μM) extracellular Ca^**2+**^ was removed and SR Ca^**2+**^ stores were depleted with CPA. Subsequently, cells were reintroduced to extracellular Ca^**2+**^ and CPA, resulting in [Ca^**2+**^]_**i**_ influx through store-operated channels (A). Both 1- and 2% CSE enhanced basal, as well as peak and plateau SOCE when compared to control (B-D). Results are mean +SE (n = 5 experiments; 100 individual cells analyzed per experiment); *P* < 0.001 and *P* < 0.0005 for the following comparisons: * 1% CSE compared to control; ** 2% CSE compared to control

hASM cells showed the characteristic transient rise in [Ca^2+^]_I_ levels when SR Ca^2+^ stores were depleted using 10 μM CPA in the absence of extracellular Ca^2+^, with a rapid rise in [Ca^2+^]_i_ levels following reintroduction of extracellular Ca^2+^ (Fig 4A, summary in Fig 4B). In cells not exposed to CSE, the peak of the rapid rise in [Ca^2+^]_i_ representing SOCE was 490 ± 4 nM. In cells treated overnight with 1% or 2% CSE, the magnitude of this SOCE was significantly higher by ~45% and ~100%, respectively (Fig 4B).

**Fig 5:**
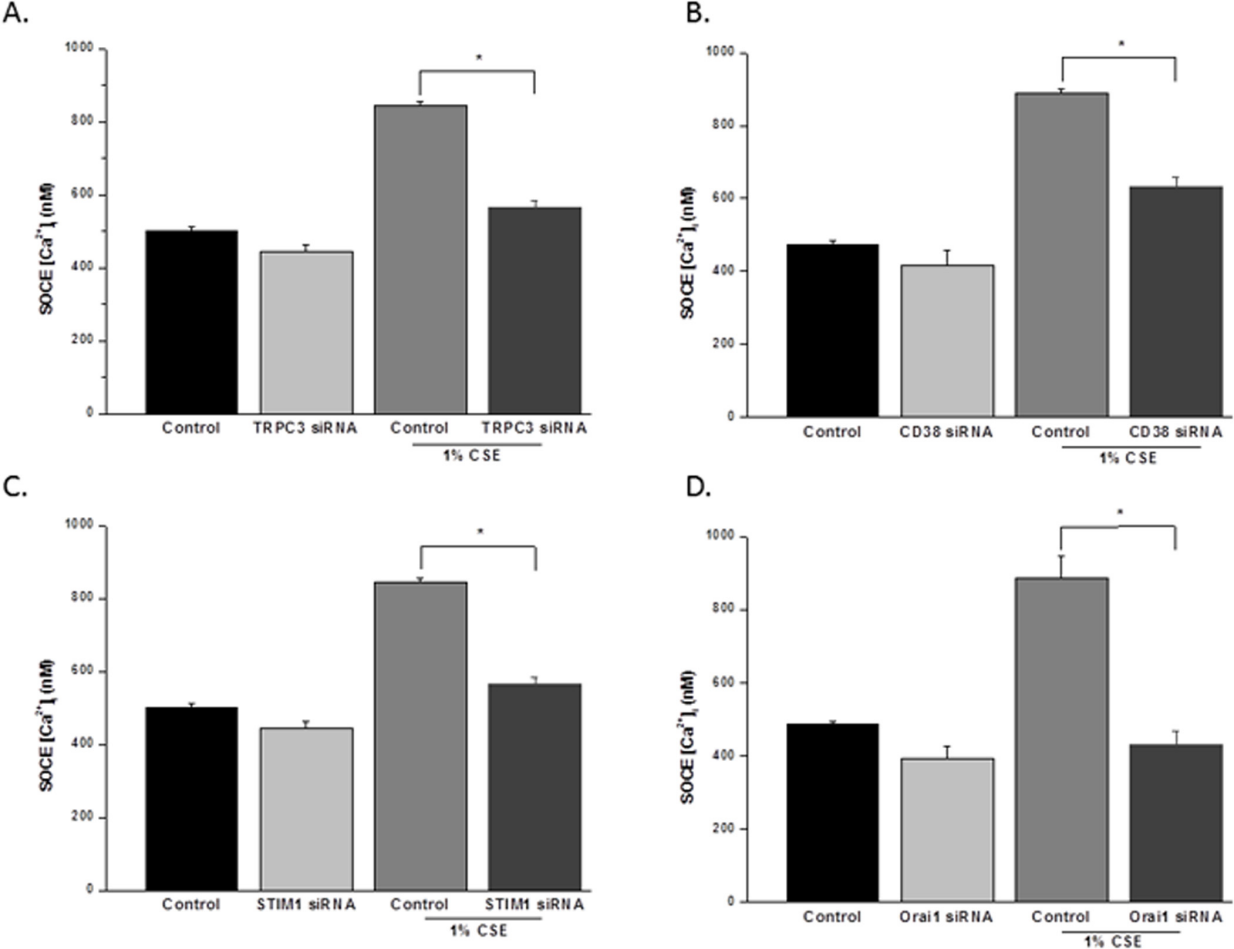
CSE enhances SOCE in hASM. A-D. Effects of TRPC3si, CD38si, STIM1si, and Orai1si on control and 1% CSE increased store-operated Ca^**2+**^ entry (SOCE) in human ASM cells. In all cell groups, depletion of sarcoplasmic reticulum Ca^**2+**^ in zero extracellular Ca^**2+**^ followed by rapid re-introduction of Ca^**2+**^ resulted in activation of SOCE. Compared to controls, 1% CSE significantly enhanced SOCE. Values are mean ± SEM. * indicates significant effect of siRNA compared to respective control (p< 0.05).

hASM cells were exposed overnight with 1% CSE or vehicle control following transfection with targeted siRNAs. CSE alone increased SOCE (as above). This effect was significantly reduced in cells transfected with siRNAs against either TRPC3 (Fig 5A), CD38 (Fig 5B), STIM1 (Fig 5C), or Orai1 (Fig 5D), compared to non-transfected cells treated with 1% CSE (P<0.05 for each siRNA effect). Among the different siRNAs, targeting of STIM1 and Orai1 was most effective in blunting CSE effects on SOCE.

### CSE enhances hASM cell proliferation

**Fig 6:**
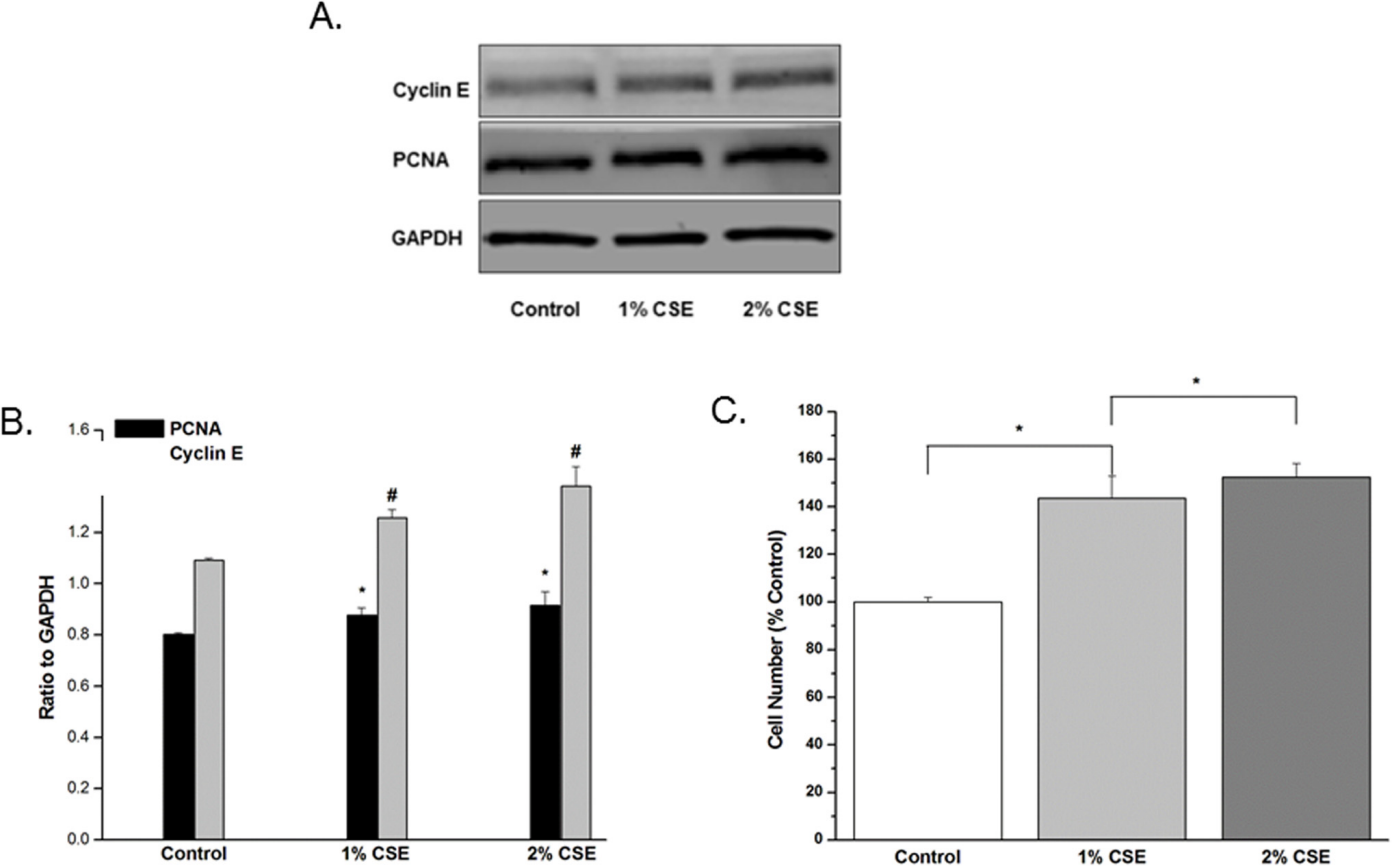
siRNAs significantly reduce CSE increased SOCE. A-C. Effect of CSE on hASM cell proliferation. A) Using fluorescent Cyquant proliferation assay, 24 hour exposure to 1- and 2% CSE induced significant cell proliferation when compared to control. Data shown as percent of control (n = 6 patients, minimum duplicate experiments per patient, * = *P* < 0.001). B) Representative Western blots demonstrating increase in both Cyclin E and PCNA which are useful markers of cell proliferation. C) Cumulative Western blot analysis on hASM cell lysates confirms proliferative effects of CSE through increased expression of Cyclin E and PCNA, known proliferative markers, with CSE exposure when compared to control samples (N = 8 patients, minimum three experiments per patient. Values are means + SE. * = *P* < 0.005 compared to control, # = *P* < 0.001 compared to control.

hASM cell proliferation was determined using fluorescent CyQuant assay. Cells treated with 1% or 2% CSE showed significant proliferation compared to control (P<0.05; Fig 6A). This pro-proliferative effect was confirmed by increased expression of Cyclin E and PCNA (Fig 6B and 6C).

### CSE increases ROS in hASM

**Fig 7:**
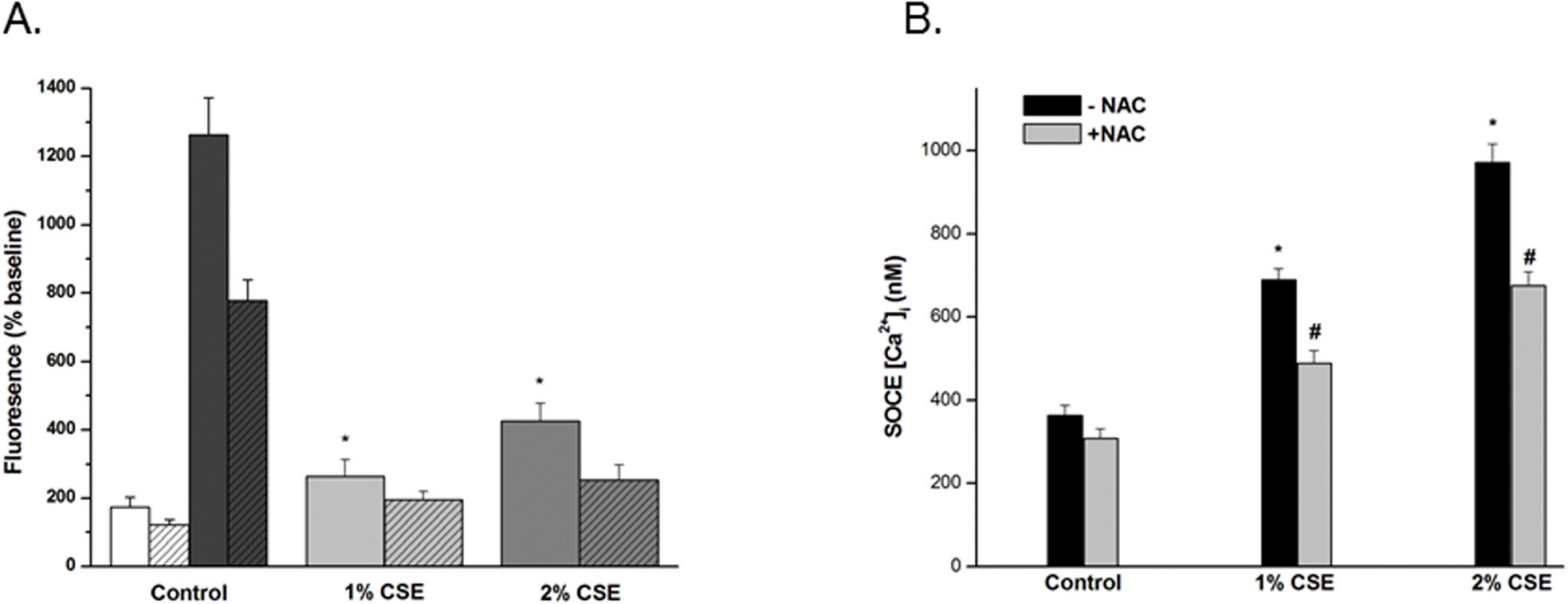
CSE enhances hASM cell proliferation. Effect of CSE on ROS production in hASM. Acute exposure to CSE (1% and 2%) increases baseline ROS generation compared to controls. Cells were loaded with CM-H2DCFDA a chloromethyl derivative of H2DCFDA which is useful as an indicator for ROS for 60 min and baseline RFU measurements were recorded. Cells were then rapidly introduced to medium only, 1-, or 2% CSE and reimaged after 15 min. CSE had immediate effects on ROS generation that were significantly greater than controls and reduced by pretreatment with NAC (hatched bars). Maximal ROS generation was elicited by H_**2**_O_**2**_ (5mM) in control cells (dark bars). (A) Values are mean ± SE. * < 0.001, n = 5–11 for each condition. Effect of ROS inhibition with NAC on SOCE. (B) Incubation with NAC (3mM) for 30mins prior to 24 h incubation with CSE + NAC (3mM). Results are mean ± SE (n = 5 experiments; 100 individual cells analyzed per experiment); *P* < 0.001 for the following comparisons: * 1% CSE compared to control; # 2% NAC compared to without NAC.

Exposure of hASM cells to 1% or 2% CSE substantially increased ROS generation within 15 min as detected by fluorescent CM-H2DCFDA dye (Fig 7). Such CSE effects were reduced by pretreatment with NAC. Maximal ROS generation was elicited by H_2_O_2_ (5mM) in control cells (Fig 7A). Conversely, inhibition of ROS via incubation with NAC (3 mM) prior to and during incubation with CSE inhibited the enhancing effect of CSE on SOCE (Fig 7B).

### CSE increases TNFα synthesis

**Fig 8:**
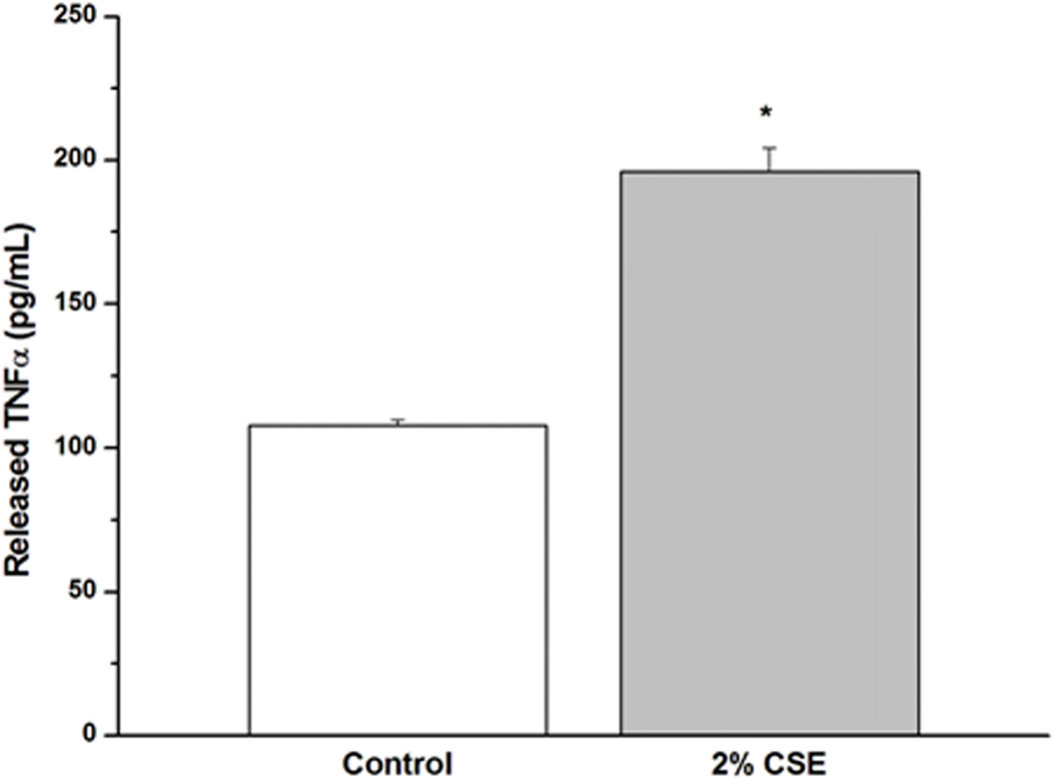
CSE increases TNFα synthesis. CSE elicits release of TNFα from hASM. To determine whether CSE exposure induces TNFα release from hASM, supernatant serum-free medium was concentrated and probed for TNFα by ELISA. Overnight exposure to 2% CSE resulted in significant TNFα release compared to non-treated controls. Values are mean ± SE. *, p < 0.001.

ELISA of supernatant serum-free medium showed that overnight exposure to 2% CSE resulted in significant TNFα release compared to non-treated controls (Fig 8).

## Discussion

Direct exposure as well as environmental cigarette smoke are prominent risk factors associated with the development, severity and exacerbations of asthma in children and COPD in adults [21,22]. Mechanisms by which cigarette smoke induces an asthma or COPD phenotype include dysfunction of ASM, the effector controlling airway caliber thus contributing to increased AHR [23]. However, the signaling and transduction events that link cigarette smoke, ASM structure and function, and AHR are less established.

Recently, several studies suggest that Ca^2+^ homeostasis is dysregulated in hASM cells from subjects with asthma and COPD. [Ca^2+^]_i_ regulation is central to ASM intrinsic force [24], as well as agonist-elicited force generation. Moreover, [Ca^2+^]_i_ regulation is an early signal in cellular proliferation. Both increased ASM force generation, as well as increased myocyte proliferation, are mechanisms contributing to increased AHR. A number of mechanisms are involved in Ca^2+^ regulation in ASM cells, among which extracellular Ca^2+^ entry plays a major role. Accordingly enhanced extracellular Ca^2+^ entry may be one mechanism for enhanced [Ca^2+^]_i_ levels. Moreover, previous studies suggest that ASM cells have enhanced proliferation due to Ca^2+^-dependent increased mitochondrial biogenesis [25] and though diminished sarco/endoplasmic reticulum Ca^2+^ ATPase (SERCA) expression [26] which may indirectly increase [Ca^2+^]_i_ levels. Overall, these data support a role for [Ca^2+^]_I_ in airway diseases such as asthma and COPD that are linked to cigarette smoke exposure. However, the role of cigarette smoke *per se* has not been established.

In the present study, we demonstrate that cigarette smoke alters [Ca^2+^]_i_ regulation in hASM cells. Specifically, we find that a central target of cigarette smoke exposure is enhanced SOCE. In this regard, first, we found increased expression of TRPC3 and CD38 in hASM tissue of bronchial airways obtained from humans with significant smoking history. The ectoenzyme CD38, the second messenger cyclic ADP ribose (cADPR), and ryanodine receptor channels play an important role in smooth muscle Ca^2+^ homeostasis. Previously, we [17] and others [27,28] have shown that *in vitro* exposure to several proinflammatory cytokines, including IL-1β, IL-13, TNF-α, and IFN-γ can increase CD38 expression, ADP-ribosyl cyclase activity, and [Ca^2+^]_i_ responses to cholinergic agonists in airway smooth muscle. The importance of CD38 for airway responsiveness has been shown in CD38 KO mice where methacholine responsiveness is significantly attenuated compared with the wild-type control mice [29]. As with CD38, we [17] and others have shown that *in vitro* exposure to proinflammatory cytokines result in TRPC3 mRNA and protein expression. Importantly, in these *in vitro* studies, CD38 and TRPC3 significantly augmented both receptor-operated calcium mobilization and SOCE [19]. The present study is the first to demonstrate increased expression of both TRPC3 and CD38 in ASM obtained from humans with COPD compared to patients without smoking history. In this regard, given the role of CD38 in mediating agonist [Ca^2+^]_i_ responses by inducing sarcoplasmic reticulum Ca^2+^ release from ryanodine receptor channels, such emptying of the SR would likely promote SR refilling via mechanisms such as SOCE. In addition, CD38 may also increase SOCE via mutual co-localization within caveolae [16,30]. The role of such interactions in the context of CSE exposure and COPD remain to be determined.

As our patient data was stratified by cigarette smoking history, we next sought to determine if short term cigarette smoke exposure of hASM cells would elicit similar data. Indeed we found that overnight incubation with 1% and 2% CSE increased TRPC3 and CD38 expression as well as the SOCE regulatory proteins Orai1 and STIM1. Both Orai1 and STIM1 proteins are molecular constituents of the capacitative calcium entry mechanism which are activated upon depletion of intracellular Ca^2+^ stores such as the sarcoplasmic reticulum. Some investigators have suggested TRPC channels associate with Orai1 and STIM1 in a dynamic ternary complex [31]. Others have defined roles for Orai1 and STIM1 as calcium channel and calcium sensor, respectively for the Ca^2+^ release activated Ca^2+^ channels (CRAC), which underlie SOCE in distinct plasma membrane domains from TRPC channels [32]. In either case CSE exposure enhances both receptor-operated calcium mobilization and SOCE. This effect is attenuated using siRNAs directed at each of these proteins, resulting in significantly reduced SOCE in CSE-exposed hASM. These findings would suggest that pharmacologic targets of CD38 and SOCE mechanistic proteins might be useful in patients with COPD and asthma.

Others have shown that CSE stimulates rat pulmonary artery smooth muscle cell proliferation via PKC-PDGFB signalling [33]. In addition CSE induces concentration dependent increase in DNA synthesis in bovine tracheal smooth muscle associated with increased cyclin D1 expression and ERK1/2 and p38 MAP kinase [34]. In the present study, we also found that CSE induces significant increases in hASM proliferation. These findings were supported by observations of increased expression of Cyclin E and PCNA.

While the mechanisms by which tobacco smoke stimulates lung cell proliferation is not known, since many extracellular signalling receptors and metalloproteinase activation are known to be redox-sensitive (19–23), we determined if CSE-generated oxygen radicals might be causal to increased proliferation. Indeed, addition of the antioxidant NAC significantly reduced CSE elicited increased hASM proliferation. Moreover, NAC significantly reduced CSE-elicited increase in SOCE suggesting that oxygen radicals underlie both enhanced [Ca^2+^]_i_ mobilization pathways and proliferation pathways. This finding is consistent with many studies which have demonstrated regulation of Ca^2+^ influx and Ca^2+^ currents by thiol modifying agents. Recently, others [35] have shown that exposure of cells to ROS may interact with cysteine residues of Orai1 and STIIM1/2 to enhance SOCE. Whether the dominate effect of ROS on SOCE occurs by effecting transcriptional regulation and, or oxidation modification of Orai 1, STIM1/2, or TRPC3 is yet to be determined.

In addition to ROS, another mechanism to consider for increased cell proliferation is SOCE itself. The physiologic role of increased SOCE in hASM, beyond increased Ca^2+^ influx is still under investigation [36]. In excitable cells, increased SOCE reduces the trigger threshold of IP3 and ryanodine signals to elicit increased [Ca^2+^]_i._ Thus, it is likely that increased SOCE would elicit AHR in clinical states of COPD and asthma. For example, YM-58483/BTP-2, a SOCE inhibitor significantly suppresses AHR in a guinea pig ovalbumin-induced bronchoconstriction model [37]. Also, there is strong evidence that SOCE is important for cell division, likely mediating cell proliferation by influencing Ca^2+^ homeostasis in the SR. Changes in levels of SR calcium are closely tied to nuclear Ca^2+^ homeostasis where the Ca^2+^ signal is necessary to induce expression of many immediate-early response genes, such as *c-Jun*, *c-Fos*, and *CRE* [38]. For example, the level of expression of different TRP proteins correlates with cell proliferation, in pulmonary artery endothelial cells [39] and in pulmonary artery smooth muscle cells [40], where TRPC proteins are mediate SOCE. Moreover, STIM1/Orai1-mediated SOCE is involved in ASM proliferation [41]. Here, our findings are in agreement with Sweeney et al. who proposed SOCE may mediate bronchial contraction and proliferation [42].

Finally, we found that CSE exposure enhances TNFα secretion from hASM cells. hASM cells can produce TNFα. Moreover, we have previously shown that TNFα incubation increases CD38, and TRPC3 expression and SOCE. Thus, it is possible that CSE-induced AHR involves TNFα synthesis by hASM resulting in autocrine/paracrine effects in the airway. There is currently no information on TNFα synthesis by hASM, but given the increasingly-recognized synthetic role of ASM, e.g. for cytokines such as IL-6 and IL-8, this is not surprising. What is less clear, and will need to be examined in future studies, is the autocrine functional relevance of TNFα synthesis by hASM.

In summary, we have demonstrated that CSE exposure of hASM cells alters [Ca^2+^]_i_ regulation, including enhanced basal [Ca^2+^]_i_, agonist-elicited [Ca^2+^]_i_, and SOCE. These findings correlate with CSE-elicited increase in expression of CD38, TRPC3, Orai1 and STIM1. siRNAs directed against these calcium regulation proteins attenuated the CSE effect on [Ca^2+^]_i_ mobilization. Moreover, CSE increases hASM SOCE in an ROS-dependent manner. CSE also increases hASM proliferation and autocrine TNFα secretion. Taken together, these calcium regulating proteins may be targets to attenuate AHR and airway smooth muscle proliferation in airway disorders such as asthma and COPD. We propose a model in which CSE, via ROS, enhances ASM SR Ca^2+^ release and activation of SOCE, as well as proliferation and cytokine synthesis leading to a phenotype of increased AHR.
